# Requirements for human‐induced pluripotent stem cells

**DOI:** 10.1111/cpr.13182

**Published:** 2022-01-26

**Authors:** Ying Zhang, Jun Wei, Jiani Cao, Kehua Zhang, Yaojin Peng, Hongkui Deng, Jiuhong Kang, Guangjin Pan, Yong Zhang, Boqiang Fu, Shijun Hu, Jie Na, Yan Liu, Lei Wang, Lingmin Liang, Huanxin Zhu, Yu Zhang, Zi‐Bing Jin, Jie Hao, Aijin Ma, Tongbiao Zhao, Junying Yu

**Affiliations:** ^1^ Nuwacell Biotechnologies Co., Ltd. Hefei City China; ^2^ Chinese Society for Stem Cell Research Shanghai China; ^3^ Zephyrm Biotechnologies Co., Ltd. Beijing China; ^4^ State Key Laboratory of Stem Cell and Reproductive Biology Institute for Stem Cell and Regeneration Institute of Zoology National Stem Cell Resource Center Chinese Academy of Sciences Beijing China; ^5^ Beijing Institute for Stem Cell and Regenerative Medicine Beijing China; ^6^ Institutes for Biological Products Control Cell Collection and Research Center National Institutes for Food and Drug Control Beijing China; ^7^ Peking University Stem Cell Research Center and Cell Biology Department Peking University Health Science Center Beijing China; ^8^ Frontier Science Center for Stem Cell Research National Stem Cell Translational Resource Center School of Life Sciences and Technology Tongji University Shanghai China; ^9^ Guangzhou Institute of Biomedicine and Health Chinese Academy of Sciences Guangzhou China; ^10^ HHLIFE Company Inc Shenzhen China; ^11^ National Institute of Metrology Beijing China; ^12^ Department of Cardiovascular Surgery of The First Affiliated Hospital & Institute for Cardiovascular Science Medical College Soochow University Suzhou China; ^13^ Center for Stem Cell Biology and Regenerative Medicine School of Medicine Tsinghua University Beijing China; ^14^ Institute for Stem Cell and Neural Regeneration School of Pharmacy Nanjing Medical University Nanjing China; ^15^ Beijing Institute of Ophthalmology Beijing Tongren Hospital Capital Medical University Beijing China; ^16^ Beijing Technology and Business University Beijing China

**Keywords:** human‐induced pluripotent stem cells, quality control, standard

## Abstract

‘Requirements for Human‐Induced Pluripotent Stem Cells’ is the first set of guidelines on human‐induced pluripotent stem cells in China, jointly drafted and agreed upon by experts from the Chinese Society for Stem Cell Research. This standard specifies the technical requirements, test methods, and instructions for use, labeling, packaging, storage, transportation, and waste handling for human‐induced pluripotent stem cells, which apply to the production and quality control of human‐induced pluripotent stem cells. It was released by the Chinese Society for Cell Biology on 9 January 2021 and came into effect on 9 April 2021. We hope that the publication of these guidelines will promote institutional establishment, acceptance, and execution of proper protocols and accelerate the international standardization of human‐induced pluripotent stem cells for applications.

## 1 SCOPE

This document specifies the technical requirements of human‐induced pluripotent stem cells and the requirements for the test methods, instructions for use, labeling, packaging, storage, transportation, and waste handling.

This standard applies to the production and testing of human‐induced pluripotent stem cells. All the citations in the text can be found in supplementary material.

## 2 NORMATIVE REFERENCES

The following documents constitute an indispensable part of this standard through normative reference. For dated references, only the edition cited applies. For undated references, only the latest edition (including all amendments) applies.

*GB/T6682* *Water for analytical laboratory use—specification and test methods*

*WS* *213 Diagnosis for hepatitis C*

*WS* *273 Diagnosis for syphilis*

*WS* *293 Diagnosis for HIV/AIDS*

*WS* *299 Diagnostic criteria for viral hepatitis B*

*T/CSCB* *0001 General requirements for stem cells*

*T/CSCB* *0002 Human embryonic stem cell*

*Pharmacopoeia* *of the People’s Republic of China*

*National* *Guide to Clinical Laboratory Procedures*

## 3 TERMS AND DEFINITIONS

For the purposes of this document, the terms and definitions in T/CSCB 0001, T/CSCB 0002, and the following terms and definitions apply.

### 3.1 Human‐induced pluripotent stem cell

These stem cells are generated from human somatic cells by reprogramming and having the ability to self‐renew indefinitely and differentiate into all derivatives of three germ layers including ectoderm, mesoderm, and endoderm.

### 3.2 Reprogramming

It is the process to generate human‐induced pluripotent stem cells by transgene expression, chemical treatment, or epigenetic modifications, etc.

## 4 ABBREVIATIONS

DNA—deoxyribonucleic acid.

EBV—Epstein‐Barr virus.

HBV—hepatitis B virus.

HCMV—human cytomegalovirus.

HCV—hepatitis C virus.

hiPSC—human‐induced pluripotent stem cell.

HIV—human immunodeficiency virus.

HTLV—human T‐lymphotropic virus.

PCR—polymerase chain reaction.

STR—short tandem repeat.

The following abbreviations apply to this document.

TP—*Treponema pallidum*.

## 5 TECHNICAL REQUIREMENTS

### 5.1 Source materials and ancillary materials

5.1.1. The raw materials, reagents, consumables and other ancillary materials, and/or supplies (e.g., gases) shall meet the requirements of T/CSCB 0001.

5.1.2. To ensure the well‐being of the donor and the safety of the donated cells, the process for donor evaluation and screening, cell collection, transportation, and receipt shall be standardized.

5.1.3. The donors shall be screened for HIV, HBV, HCV, HTLV, EBV, HCMV, and TP, and the results shall be documented.

### 5.2 Critical quality attributes

#### 5.2.1 Cell morphology

Cells grown under 2D conditions shall form compact colonies with clear edges, exhibit uniform morphology with a high nucleus‐to‐cytoplasm ratio, and have tight junctions between cells.

#### 5.2.2 Chromosome karyotype

The normal karyotype shall be 46, XY, or 46, XX.

#### 5.2.3 Cell viability

Cell viability shall be ≥90% before cryopreservation, and ≥60% post‐thaw.

#### 5.2.4 Cell markers

The expression of at least two of the cell surface markers SSEA3, SSEA4, TRA‐1‐60, and TRA‐1‐81 shall be ≥70.0% of the cell population, and the expression of the intracellular marker OCT4 and NANOG shall be ≥70.0% of the cell population.

#### 5.2.5 Teratoma formation

Teratoma formation shall be able to form teratomas with derivatives from all three germ layers in vivo.

#### 5.2.6 Microorganisms

Microorganisms shall be negative for fungi, bacteria, mycoplasma, HIV, HBV, HCV, HTLV, EBV, HCMV, and TP.

### 5.3 Process control

#### 5.3.1 Cell authentication

The identity of hiPSCs shall match that of donor cells by STR analysis.

#### 5.3.2 Reprogramming method

The method used for hiPSC reprogramming shall be documented.

#### 5.3.3 Exogenous reprogramming genes

The expression and genomic integration of exogenous reprogramming genes shall be tested and documented.

Note: if hiPSCs are used as the raw material for manufacturing cell products for clinical applications, the test results for the expression and genomic integration of the exogenous reprogramming genes shall be negative.

## 6 TEST METHODS

### 6.1 Cell morphology

Cell morphology observes the morphology of cells grown under 2D conditions in vitro using an inverted phase‐contrast microscope.

### 6.2 Chromosome karyotype

The method in the *Pharmacopoeia of the People’s Republic of China* (the provision “Preparation and quality control of animal cell substrates used in manufacturing and analysis of biological products”) shall be followed.

### 6.3 Cell viability

The method in Appendix A shall be followed.

### 6.4 Cell markers

The method in Appendix B shall be followed.

### 6.5 Exogenous reprogramming genes

The method in Appendix C shall be followed.

### 6.6 Teratoma formation

The method in Appendix D shall be followed.

### 6.7 Microorganisms

#### 6.7.1 Fungi

The method in the *Pharmacopoeia of People’s Republic of China* (section 1101—Sterility tests) shall be followed.

#### 6.7.2 Bacteria

The method in the *Pharmacopoeia of People’s Republic of China* (section 1101—Sterility tests) shall be followed.

#### 6.7.3 Mycoplasma

The method in the *Pharmacopoeia of People’s Republic of China* (section 3301—Mycoplasma tests) shall be followed.

#### 6.7.4 HIV

The nucleic acid method in WS 293 shall be followed.

#### 6.7.5 HBV

The nucleic acid method in WS 299 shall be followed.

#### 6.7.6 HCV

The nucleic acid method in WS 213 shall be followed.

#### 6.7.7 HTLV

The nucleic acid method in the *National Guide to Clinical Laboratory Procedures* shall be followed.

#### 6.7.8 EBV

The nucleic acid method in the *National Guide to Clinical Laboratory Procedures* shall be followed.

#### 6.7.9 HCMV

The nucleic acid method in the *National Guide to Clinical Laboratory Procedures* shall be followed.

#### 6.7.10 TP

The nucleic acid method in WS 273 shall be followed.

#### 6.7.11 Adventitious viruses

The method in the *People’s Republic of China Pharmacopoeia* (section 3302—Adventitious virus tests) shall be followed.

## 7 INSPECTION RULES

### 7.1 Sampling method and quantity

7.1.1. Cells produced in the same production cycle with the same production line, the same source, the same passage number, and the same production process are considered to be the same batch.

7.1.2. The three smallest units of packaging shall be randomly sampled from the same batch^1^.

### 7.2 Quality inspection and release

7.2.1. Each batch of products shall be subject to quality inspection before release, and inspection reports shall be attached.

7.2.2. The quality inspection shall include all items listed in Section 5.2.

### 7.3 Review inspection

Review inspection shall be performed by professional cytological testing organizations/laboratories as necessary.

### 7.4 Decision rules

7.4.1. Products that pass all requirements in Section 5.2 for the quality inspection for release are considered to be qualified. Products that fail to pass one or more requirements in Section 5.2 for the quality inspection for release are considered to be unqualified.

7.4.2. Products that pass all requirements in Section 5.2 for the quality review inspection are considered to be qualified. Products that fail to pass one or more requirements in Section 5.2 for the review inspection are considered to be unqualified.

## 8 INSTRUCTIONS FOR USE

The instructions for use shall include, but not limited to:

- Product name;
- Passage number;
- Cell numbers;
- Reprogramming method;
- Test results of residual exogenous reprogramming genes;
- Production date;
- Batch number;
- Production organization;
- Storage conditions;
- Shipping conditions;
- Operation manual;
- Executed standard number;

Note: according to what standards are the cells produced

m) Manufacturing address;

Note: alternatively refers to the laboratory where the cells were derived.

n) Contact information;
o) Postal code;
p) Matters that need attention.

Note: upon user’s requirement, endotoxin test results can be provided.

## 9 LABELS

The label shall include but not limited to:

- Product name;
- Passage number;
- Cell numbers;
- Batch number;
- Production organization;
- Production date.

## 10 PACKAGE, STORAGE, AND TRANSPORTATION

### 10.1 Package

The material and container selected shall not interfere with the critical quality attributes of hiPSCs.

### 10.2 Storage

10.2.1. T/CSCB 0001 and T/CSCB 0002 shall be followed.

10.2.2. Cryopreserved cell products shall be stored at temperatures below −130°C.

### 10.3 Transportation

10.3.1. T/CSCB 0001 and T/CSCB 0002 shall be followed.

10.3.2. Cryopreserved cell products shall be transported with dry ice or below −130°C. Non‐frozen cell products are recommended to transport at 2–8°C.

## 11 WASTE HANDLING

Waste generated during the production process and quality inspection tests shall be handled according to the requirements of T/CSCB 0001.

## CONFLICT OF INTEREST

The authors declare no competing financial interests.

## AUTHOR CONTRIBUTIONS

JY and TZ contributed to conception and design. YZ (Ying Zhang) drafted and revised the manuscript. AM, KZ, HD, JK, ZJ, GP, JH, YZ (Yong Zhang), BF, SH, JN, YL, JC, LW, and HZ critically read and revised the manuscript.

## Supporting information

Supplementary Material

## Data Availability

Data are available upon request.

## ENDNOTES

^1^
A batch mean products produced according to a single manufacturing order during the same cycle of manufacture.

## APPENDIX A

### Cell counting and viability test

#### A.1 Instruments

A.1.1 Microscope

A.1.2 Neubauer hemocytometer

#### A.2 Reagents

Unless otherwise specified, all reagents shall be analytical pure. The water used in all tests shall be deionized.

A.2.1 Phosphate‐buffered saline: pH 7.4

A.2.2 Trypan blue solution: the concentrated stock solution is diluted to 0.4% (W/V) with phosphate‐buffered saline (A.2.1) for use.

#### A.3 Procedure

A.3.1 Preparing single‐cell suspension^2^

Collect the cells to be tested and resuspend the cells in phosphate‐buffered saline (A.2.1).

A.3.2 Trypan blue staining

Dilute the cell suspension (A.3.1) in trypan blue solution (A.2.2) at 1:1 and mix well.

A.3.3 Cell counting

Put coverslips on each chamber of a clean hemocytometer (A.1.2). Transfer 10 µL trypan blue/cell suspension (A.3.2) to the edge of the coverslip, allowing the cell suspension to fill the chambers under the coverslip without over‐ or under‐fill. Repeat it with the second chamber. Let stand for 30 seconds, and count all cells (stained and unstained) and the stained cells in each chamber under a microscope (A.1.1).

Repeat steps A.3.2 to A.3.3 once.

#### A.4 Viability calculation

The cell viability can be calculated with the following formula Equation (1) ( A.1):

(1)
$$S=\left(M‐D\right)/M*100\%.\left(A.1\right)$$

where

*S*—cell viability

*M*—total cell number

*D*—stained cell number

Calculate the average viability of two repeats. This result is recorded as the viability of cells.

#### A.5 Precision

Under the same conditions, the absolute difference between the results of the two repeats shall not exceed 10% of their arithmetic mean.

## APPENDIX B

### Flow cytometry analysis of cell markers

#### B.1 Instruments

B.1.1 Flow cytometer

B.1.2 Horizontal centrifuge

B.1.3 Electronic balance

#### B.2 Reagents

All reagents in this method shall be analytical pure. Unless otherwise specified, the water used in all tests shall be the level 1 water as specified in GB/T 6682.

B.2.1 Sodium chloride (NaCl): analytical pure

B.2.2 Potassium chloride (KCl): analytical pure

B.2.3 Disodium hydrogen phosphate anhydrous (Na_2_HPO_4_): analytical pure

B.2.4 Potassium dihydrogen phosphate anhydrous (KH_2_PO_4_): analytical pure

B.2.5 Paraformaldehyde (PFA): 95% purity

B.2.6 Sodium hydroxide (NaOH): analytical pure

B.2.7 Bovine serum albumin (BSA): purity ≥98%

B.2.8 Triton X‐100

B.2.9 Antibodies

B.2.10 Prepare solutions needed for flow cytometry assay using an electronic balance (B.1.3): washing buffer, fixation buffer, blocking/permeabilization buffer, and antibody dilution buffer.

#### B.3 Preparation and storage of samples

The washing buffer and fixed samples shall be stored at 2~8°C.

The fixation buffer shall be divided into aliquots, sealed, labeled, and stored according to the product instruction/manual.

The antibodies shall be stored according to their instructions/manuals.

#### B.4 Procedure

B.4.1 Sample preparation and fixation

Harvest dissociated single cells by centrifugation at 250 × *g* for 3 min. Discard the supernatant. Resuspend the cells in an appropriate volume of the fixation buffer. Place the suspension on ice for 10 min. Wash fixed cells with an appropriate volume of the washing buffer for 3–5 min using the horizontal centrifuge (B.1.2) and repeat 3–5 times.

B.4.2 Blocking and permeabilization

Resuspend the cells with blocking/permeabilization buffer. Divide the cell suspension into two equal aliquots for isotype control and test. Incubate the suspension on ice for 20 min. Wash the cells with washing buffer once.

B.4.3 Antibody staining

Dilute and stain the cells with antibodies according to their instructions/manuals.

B.4.4 Cell filtration and analysis

Resuspend the cells with the washing buffer, and filter the suspension with a 40‐µm mesh to remove possible cell clumps. Collect the filtration in a cytometry tube. Analyze the samples on a flow cytometer (B.1.1) following the user manual.

B.4.5 Gating

First, exclude events of debris, dead cells, and untargeted cell populations by drawing a gate (gate 1) according to estimated cell size (FSC) and granularity (SSC). Next, by comparing the test and isotype control, place the gate for positive staining population (gate 2) to exclude the cells not labeled with fluorescent antibodies. Isotype antibodies should be used as the negative control.

#### B.5 Analysis of results

The results of flow cytometry analysis shall be analyzed comprehensively with appropriate software following its user manual.

## APPENDIX C

### Quantitative PCR analysis of exogenous reprogramming genes

#### C.1 Instrument

Quantitative PCR system

#### C.2 Reagents

C.2.1 Commercial kit for quantitative PCR

C.2.2 Commercial genomic DNA extraction kit

C.2.3 PCR primers for the target genes

#### C.3 Procedure

C.3.1 Extract the genomic DNA from cell samples according to the user manual provided with the kit (C.2.2).

C.3.2 Perform quantitative PCR (C.1) to establish the standard curve using target gene DNA reference standard according to the user manual provided with the kit (C.2.1).

C.3.3 Perform quantitative PCR (C.1) to quantify the copies of target genes present in genomic DNA from C.3.1 according to the user manual provided with the kit (C.2.1).

C.3.4 Calculate the concentrations of the target genes using the standard curve.

## APPENDIX D

### Pluripotency test with teratoma formation assay

#### D.1 Instrument

D.1.1 Hemocytometer

D.1.2 Microscope

D.1.3 Horizontal centrifuge

D.1.4 Electrical balance

D.1.5 Embedding machine

D.1.6 Microtome

D.1.7 Tissue floatation water bath

D.1.8 Glass slides

D.1.9 Coverslips

D.1.10 Slide dryer or convection oven

D.1.11 Fume hood

#### D.2 Reagents

D.2.1 All reagents in this method shall be analytical grade. Unless otherwise specified, the water used in all tests shall be the level 1 water as specified in GB/T 6682^3^.

D.2.2 Phosphate‐buffered saline

D.2.3 Dissociation enzyme(s)^4^

D.2.4 4% Trypan blue solution

D.2.5 Absolute ethanol (C_2_H_5_OH): analytical pure

D.2.6 Xylene (C_8_H_10_): analytical pure

D.2.7 Paraffin (melting point 60℃)

D.2.8 4% Paraformaldehyde solution

D.2.9 Hematoxylin staining buffer

D.2.10 Eosin staining buffer

D.2.11 Neutral balsam

#### D.3 Procedure

D.3.1 Cell sample preparation

D.3.1.1 Cell dissociation

Following the instruction of the applied dissociation enzyme, incubate hiPSCs with the enzyme for an appropriate time. Centrifuge to collect dissociated cells in a centrifuge tube. Gently resuspend cells in normal saline. Avoid creating bubbles or cell clumps.

D.3.1.2 Cell counting

Determine viable cell concentration in the suspension using the method described in Appendix A.

D.3.2 Cell transplantation

Inject 1 × 10^6^–1 × 10^7^ hiPSCs to each 6‐ to 8‐week‐old immunodeficient mouse subcutaneously, or intramuscularly, or into the seminiferous tubule space under the albuginea of the testis, or under the renal capsule using syringes.

D.3.3 Teratoma collection and processing

D.3.3.1 Teratoma collection

Six to 10 weeks after hiPSC injection (make sure the teratoma does not exceed 15% of the bodyweight), euthanize the mice, and remove teratomas. Cut the teratoma into small pieces (with sizes below 5 × 5 × 2 mm), and fix the samples by incubating in 4% paraformaldehyde overnight at 4°C.

D.3.3.2 Paraffin sectioning and hematoxylin and eosin staining

Section the fixed samples following paraffin embedding. Perform hematoxylin and eosin staining. Observe under a microscope and take pictures for documentation.

#### D.4 Analysis of results

If derivatives of all three germ layers are observed, e.g., endoderm‐derived gut gland epithelium, mesoderm‐derived cartilage, and ectoderm‐derived neural tissues, the hiPSCs injected are considered to be pluripotent capable of differentiating into all three germ layers in vivo.

