# Supplementary material for: Requirements for human‐induced pluripotent stem cells
Source: Cell Prolif. 2022 Jan 26;55(4):e13182. doi: 10.1111/cpr.13182 (PMC9055897; doi:10.1111/cpr.13182)
Supplement: Supplementary file 1 — Supplementary Material [file CPR-55-e13182-s001.doc]

Title: Requirements for Human Induced Pluripotent Stem Cells

Supplementary Information

***Clarifications regarding:***

**1) Scope**

The main text is a translation of a standard by the Chinese Society for Cell Biology published on Jan 9, 2021, effective on Apr 9, 2021. The purpose of this standard is to provide a reliable framework for the production and testing of hiPSC products in research and development in the vision of future clinical applications. The academic research projects conducted by individual laboratories, especially human rare or genetic disease iPSC research, are not within the scope of the standard.

**2) Normative references**

Most of the normative references listed in the main text became effective by publishing as hard copies in China. Here we provide the source information in the table below should readers intend to explore further.

| GB/T6682-2008 Water for analytical laboratory use – specification and test method | Standard Book #155066.1-32760 |
| --- | --- |
| WS 213 Diagnosis for hepatitis C | http://www.nhc.gov.cn/wjw/s9491/201803/29997c16d2f24e639ab6c6f55105a9d0.shtml |
| WS 273 Diagnosis for syphilis | http://www.nhc.gov.cn/wjw/s9491/201803/5103a5425f9e47d29b91de38434b7f74.shtml |
| WS 293 Diagnosis for HIV / AIDS | http://www.nhc.gov.cn/wjw/s9491/201905/6430aa653728439c901a7340796e4723.shtml |
| WS 299 Diagnostic criteria for viral hepatitis B | http://www.nhc.gov.cn/wjw/s9491/200907/41983.shtml |
| T/CSCB 0001 General requirements for stem cells | Standard Book #155066.2-32089 |
| T/CSCB 0002 Human embryonic stem cell | Standard Book #155066.2-34099 |
| Pharmacopoeia of the People’s Republic of China | ISBN：978-7-5214-1575-9 |
| National Guide to Clinical Laboratory Procedures | ISBN：978-7-1171-9862-2 |

**3) Chromosome karyotype**

The standard applies to the production and testing of hiPSC products in research and development in the vision of future clinical applications. Therefore, the karyotype of diploid hiPSC derived from healthy donors shall be 46, XY, or 46, XX. HiPSCs with non-diploid karyotypes indeed provide a powerful tool to investigate rare or inherited human diseases. But using hiPSCs as disease models is not within the scope of the current standard.

**4) Test methods**

Given the rapid development of hiPSCs products, we do plan to provide regular updates of this standard in the future, in which the listed analytical methods are subject to revision or being replaced by validated alternatives. In addition, we plan to draft a clinical-grade iPSC standard based on the current “minimum requirement” standard when experts’ consensus is reached.

**5) References**

The format of the Chinese Society for Cell Biology group standard does not specify a reference section usually included in research articles. During the process of drafting this standard, we referred to the following scientific publications.

1. J. M. Crook *et al.*, The generation of six clinical-grade human embryonic stem cell lines. *Cell Stem Cell* **1**, 490-494 (2007).

2. M. S. Rao, N. Malik, Assessing iPSC reprogramming methods for their suitability in translational medicine. J Cell Biochem 113, 3061-3068 (2012).

3. J. P. Awe et al., Generation and characterization of transgene-free human induced pluripotent stem cells and conversion to putative clinical-grade status. Stem Cell Res Ther 4, 87 (2013).

4. P. A. Goh et al., A systematic evaluation of integration free reprogramming methods for deriving clinically relevant patient specific induced pluripotent stem (iPS) cells. PLoS One 8, e81622 (2013).

5. B. A. Baghbaderani et al., cGMP-Manufactured Human Induced Pluripotent Stem Cells Are Available for Pre-clinical and Clinical Applications. Stem Cell Reports 5, 647-659 (2015).

6. M. A. Canham et al., The Molecular Karyotype of 25 Clinical-Grade Human Embryonic Stem Cell Lines. Sci Rep 5, 17258 (2015).

7. T. M. Schlaeger et al., A comparison of non-integrating reprogramming methods. Nat Biotechnol 33, 58-63 (2015).

8. I. Garitaonandia et al., Neural Stem Cell Tumorigenicity and Biodistribution Assessment for Phase I Clinical Trial in Parkinson's Disease. Sci Rep 6, 34478 (2016).

9. L. A. Wiley et al., cGMP production of patient-specific iPSCs and photoreceptor precursor cells to treat retinal degenerative blindness. Sci Rep 6, 30742 (2016).

10. Q. Gu et al., Accreditation of Biosafe Clinical-Grade Human Embryonic Stem Cells According to Chinese Regulations. Stem Cell Reports 9, 366-380 (2017).

11. L. A. Wiley et al., Generation of Xeno-Free, cGMP-Compliant Patient-Specific iPSCs from Skin Biopsy. Curr Protoc Stem Cell Biol 42, 4A 12 11-14A 12 14 (2017).

12. S. Sullivan et al., Quality control guidelines for clinical-grade human induced pluripotent stem cell lines. Regen Med 13, 859-866 (2018).

13. B. Alvarez-Palomo et al., Adapting Cord Blood Collection and Banking Standard Operating Procedures for HLA-Homozygous Induced Pluripotent Stem Cells Production and Banking for Clinical Application. J Clin Med 8, (2019).

14. Y. Avior, K. Eggan, N. Benvenisty, Cancer-Related Mutations Identified in Primed and Naive Human Pluripotent Stem Cells. Cell Stem Cell 25, 456-461 (2019).

15. A. Haase et al., GMP-compatible manufacturing of three iPS cell lines from human peripheral blood. Stem Cell Res 35, 101394 (2019).
